# Dimethyl Fumarate Protects against Lipopolysaccharide- (LPS-) Induced Sepsis through Inhibition of NF-*κ*B Pathway in Mice

**DOI:** 10.1155/2023/5133505

**Published:** 2023-10-05

**Authors:** He Fang, Xingtong Wang, Mahendra Damarla, Rongju Sun, Qingli He, Ruojing Li, Pengfei Luo, Jun O. Liu, Zhaofan Xia

**Affiliations:** ^1^Department of Burn Surgery, The First Affiliated Hospital of Naval Medical University, 168 ChangHai Road, Yangpu District, Shanghai 200433, China; ^2^Department of Pharmacology and Molecular Sciences, School of Medicine, Johns Hopkins University, 725 North Wolfe Street, Baltimore, MD 21205, USA; ^3^Research Unit of Key Techniques for Treatment of Burns and Combined Burns and Trauma Injury, Chinese Academy of Medical Sciences, 168 ChangHai Road, Yangpu District, Shanghai 200433, China; ^4^Department of Burns and Plastic Surgery, The Fourth Medical Center of General Hospital, The People's Liberation Army, Beijing 100048, China; ^5^Department of Pulmonary and Critical Care Medicine, The Johns Hopkins Hospital, 1830 E. Monument Street, Baltimore, MD 21287, USA; ^6^Department of Emergency, The Eighth Medical Center, General Hospital of PLA, Beijing 100853, China; ^7^Innovation Research Institute of Traditional Chinese Medicine, Shanghai University of Traditional Chinese Medicine, 1200 Cailun Road, Shanghai 201203, China

## Abstract

Sepsis is one of the most severe complications and causes of mortality in the clinic. It remains a great challenge with no effective treatment for clinicians worldwide. Inhibiting the release of proinflammatory cytokines during sepsis is considered as an important strategy for treating sepsis and improving survival. In the present study, we have observed the effect of dimethyl fumarate (DMF) on lipopolysaccharide- (LPS-) induced sepsis and investigated the possible mechanism. By screening a subset of the Johns Hopkins Drug Library, we identified DMF as a novel inhibitor of nitric oxide synthesis in LPS-stimulated RAW264.7 cells, suggesting that DMF could be a potential drug to treat sepsis. To further characterize the effect of DMF on LPS signaling, TNF-*α*, MCP-1, G-CMF, and IL-6 expression levels were determined by using cytokine array panels. In addition, an endotoxemia model with C57BL/6 mice was used to assess the in vivo efficacy of DMF on sepsis. The survival rate was assessed, and HE staining was performed to investigate histopathological damage to the organs. DMF was found to increase the survival of septic mice by 50% and attenuate organ damage, consistent with the reduction in IL-10, IL-6, and TNF-*α* (inflammatory cytokines) in serum. In vitro experiments revealed DMF's inhibitory effect on the phosphorylation of p65, I*κ*B, and IKK, suggesting that the primary inhibitory effects of DMF can be attributed, at least in part, to the inhibition of phosphorylation of I*κ*B*α*, IKK as well as nuclear factor-*κ*B (NF-*κ*B) upon LPS stimulation. The findings demonstrate that DMF dramatically inhibits NO and proinflammatory cytokine production in response to LPS and improves survival in septic mice, raising the possibility that DMF has the potential to be repurposed as a new treatment of sepsis.

## 1. Introduction

Inflammation, an initial host immune reaction, is critical for the host's defense against microbial infection, injury, and stress [1–3]. As such, it plays an essential role in protecting the host from bacterial and viral infections. Excessive or inappropriate inflammatory responses, on the other hand, frequently contribute to the pathology of numerous illnesses, including sepsis, cancer, allergies, and asthma [4–7]. Sepsis can be caused by a wound, bacteria, or bacterial toxins like lipopolysaccharide (LPS), which is considered caused by the hosts' overreaction to an inflammatory agent [8, 9]. Preventing and treating sepsis requires early control of inflammatory overreaction [10, 11].

Macrophages are an essential component involved in the pathophysiology of the overreaction of inflammatory responses [12, 13]. LPS is a glycolipid present in Gram-negative bacteria's outer membrane and activates macrophages through binding to toll-like receptor 4 (TLR-4) [14]. Activated macrophages produce inflammatory cytokines such as interleukin-6 (IL-6), tumor necrosis factor-*α* (TNF-*α*), interleukin-1*β* (IL-1*β*), as well as inflammatory mediators like nitric oxide (NO) [15, 16].

**Figure 1:**
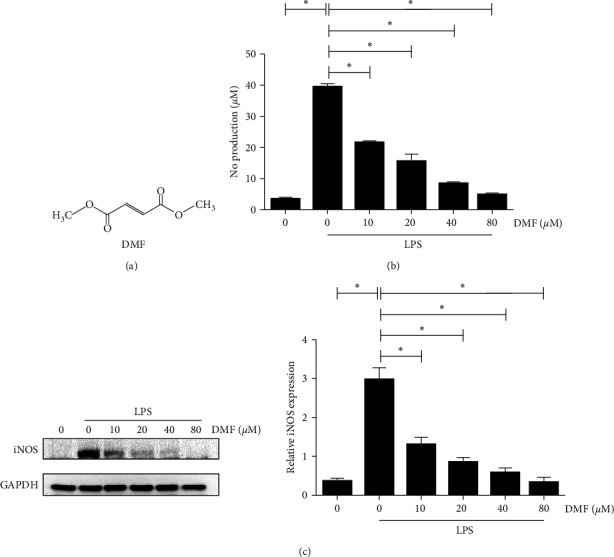
DMF effects on LPS-induced NO synthesis and iNOS expression. (a) Chemical structure of DMF. (b, c) Inhibition of LPS-stimulated NO synthesis and iNOS expression by DMF. LPS or LPS as well as various concentrations of DMF (10, 20, 40, and 80 *µ*M) were used to treat RAW264.7 cells. On x-aixs, the first 0 corresponds to the blank group and the second 0 corresponds to the LPS stimulation group (without DMF). NO synthesis was evaluated by Griess reagent and expression of iNOS was performed by western blot. The protein levels of iNOS were statistical analyzed by semi-quantitation method. The values represent the mean ± SD of three separate experiments and Student's *t*-test was utilized for evaluating the differences in the mean values.  ^*∗*^Suggests significant difference between the indicated two groups (*P* < 0.05).

To identify new inhibitors of LPS-induced production of proinflammatory mediators, we set up a cell-based screen using NO as a readout. Using this assay, we screened the Johns Hopkins Drug Library. Among the most interesting hits is dimethyl fumarate (DMF, Figure 1(a)). DMF was initially approved for the treatment of psoriasis in Germany in 1994 under the trade name Fumaderm [17]. In March 2013, DMF in a new formulation, BG-12 (brand name Tecfidera), was approved as first-line therapy for multiple sclerosis (MS) by the US Food and Drug Administration (FDA) [18]. The exact mechanism of action of DMF remains incompletely understood. We further characterized the likely molecular mechanism of inhibition of LPS signaling by DMF in vitro and determined its effect on LPS-induced sepsis in vivo.

## 2. Materials and Methods

### 2.1. Chemicals and Reagents

Sigma Chemical Co. (St. Louis, MO) provided DMF, LPS (*Escherichia coli* 055:B5), and dimethylsulfoxide (DMSO). Invitrogen Gibco (Grand Island, NY) provided penicillin, streptomycin, fetal bovine serum (FBS), and Dulbecco's modified Eagle's medium (DMEM). Polyclonal antibodies against phospho-p44/42 MAPK (Erk1/2), IKK*α*, and I*κ*B*α*, monoclonal antibodies against phosphor-IKK*α*/*β*, phosphor-I*κ*B*α*, NF-*κ*b p65, and phosphor-NF-*κ*B p65 were supplied by the Cell Signaling Technology Inc. (Beverly, MA). Polyclonal antibodies against GAPDH, iNOS, and ERK1 were bought from Santa Cruz Biotechnology (CA). GE Healthcare (Buckinghamshire, UK) provided goat–mouse secondary antibodies as well as HRP-conjugated anti-rabbit. We bought polyvinylidenefluoride (PVDF) membrane from Whatman GmbH (Germany). Thermo Fisher Scientific Inc. (Pierce, Rockford) provided Pierce ECL western blotting substrate. The Cytometric Bead Array (CBA) Mouse Cytokine Kit was purchased from BD (Becton, Dickinson and Company, USA). The rest of the chemicals were of reagent grade.

### 2.2. Cell Culture and Model Animals

The American Type Culture Collection (ATCC) supplied the murine macrophage cell line RAW264.7, cultured in DMEM with 10% heat-inactivated FBS, penicillin (100 U/ml), as well as streptomycin (100 *μ*g/ml). The cells were incubated at 37°C in a humidified environment containing 5% CO_2_ and subcultured every 2 days. The male C57Bl/6 mice (20–25 g) were obtained from the Experimental Animal Center of NMU in China and Jackson Laboratory in the USA. They were kept in a controlled environment (23 ± 3°C, 50 ± 10% humidity, and 12 hr day/night rhythm) and fed standard laboratory chow. Animal experiments were approved by Johns Hopkins University Animal Care and Use Committee (ACUC) in accordance with the Guide for Care and Use of Laboratory Animals published by U.S. National Institutes of Health (NIH).

### 2.3. Determination of NO Synthesis

The amount of nitrite accumulated in the incubation media was used to determine NO synthesis. RAW264.7 cells were plated at a density of 5 × 10^5^ cells/well in 6-well plates. After 24 hr of incubation, the cells were treated for 18 hr with 0.1 *µ*g/ml LPS supplemented with different concentrations of DMF. An aliquot of 50 *µ*l of culture supernatant was combined with 50 *µ*l of Griess reagent (1% sulfanilamide and 0.1% naphthylethylenediamine dihydrochloride in 2.5% phosphoric acid) and then incubated for 15 min at room temperature. Then, the absorbance at 540 nm was measured using OPTIMA plate reader. A sodium nitrite standard curve was used to calculate the nitrite concentrations.

### 2.4. Western Blot

RAW264.7 cells were plated at a density of 5 × 10^5^ cells/well in 6-well plates. After incubation for 24 hr, before being stimulated with 0.1 *µ*g/ml LPS for 15 min, we pretreated the cells with various concentrations of DMF for 15 min. After discarding the supernatant, the cells were washed with cold PBS twice. Subsequently, lysis buffer (50 mM Tris·HCl, pH 7.4, 150 mM NaCl, 1% Triton X-100 1% sodium deoxycholate, 0.1% SDS, 20 mM *β*-glycerophosphate, 1 mM phenylmethylsulfonylfluoride, 2 mM *p*-nitrophenylphosphate, and 1 : 50 protease inhibitor) was used to lyse the cells for 30 min on ice. The lysates were resolved by 10% SDS-PAGE and proteins were transferred into PVDF membranes. Then, we treated the membranes with specified primary antibodies overnight at 4°C after blocking with 5% BSA in TBST buffer at room temperature for an hour. The membranes were rinsed three times with TBST before being incubated for 1 hr with HRP secondary antibodies at room temperature. Chemiluminescence substrates were used to detect the HRP signal on the membrane.

### 2.5. Endotoxaemia Model

Sixty-six male C57BL/6 mice (7–8 weeks old) were included in the study. To investigate the survival rate, 30 mice were randomly divided into three groups using the random number table (*n* = 10 per group): control group, LPS group and DMF group. The mice in control group were given neither LPS nor DMF, and the mice in LPS group were intraperitoneally injected with LPS (20 mg/kg) to induce the endotoxemia model. The mice in DMF group were orally given DMF (dose: 30 mg/kg body weight) in 0.08% methocel as carrier solution twice a day for two consecutive days, then LPS (20 mg/kg) was intraperitoneally administered to the mice in the DMF group. The death time of these mice was recorded, and the survival rate was determined using a log-rank statistical approach. To investigate the DMF effects on the organ and the blood sampling in LPS-induced septic mice, 36 mice were randomly assigned to three groups (*n* = 12 per group): control group, LPS group, and DMF group. The treatment methods of each group were the same as the above-mentioned investigation experiments of survival rate. The lung, kidney, and liver samples were obtained after 24 hr of establishing the sepsis model, and blood samples were collected in different groups at indicated hours. The tissues were fixed in 10% formalin for 24 hr, and the fixed tissues were embedded in paraffin for H&E staining. H&E-stained 5-*µ*m-thick slices were imaged using a fluorescence microscope (Leica, German). Serum samples were stored at −80°C prior to analysis. All mice were anesthetized via intraperitoneal injection with 1% sodium pentobarbital (50 mg/kg). The mice were euthanized with 100% carbon dioxide.

The injury scores were determined according to a previously established method [19]. Alveolar congestion, hemorrhage, neutrophil infiltration into the vessel wall or airspace, and the alveolar wall thickness/formation of the hyaline membrane were used to calculate the lung injury score. Hemorrhage, inflammatory cell infiltration, and necrosis of hepatocytes in the liver tissue were used to calculate the liver injury score. The kidney injury score was as per the renal tubule injuries as well as the shrunk glomerulus. Two independent pathologists blindly assessed histopathological abnormalities in the lung, liver, and kidney samples.

### 2.6. Mouse Cytokine Array Panel

Each array unit was filled with 100 *μ*l culture medium supernatant, and each membrane needs to be sealed with 2 ml of the corresponding array buffer and incubated on the shaking table for 1 hr. The dilution of the sample was done in the matching array buffer with 1.5 ml volume per hole, and the sample was then incubated for an hour at room temperature with the diluted test antibody. The appropriate array unit was then introduced, followed by overnight incubation at 4°C with the array protein membrane. After three washes with wash buffer, streptavidin-HRP was added, followed by a 0.5 hr of incubation and shaking of the membrane at room temperature. The film was washed thrice using wash buffer, and then each hole was filled with 1 ml of chemical reagent. Subsequently, we developed the color using a chemiluminescence imager. Professional software was used to examine the data.

### 2.7. Mouse Cytometric Bead Array

The mixed capture beads were vortexed before 50 *μ*l of bead suspension was aliquoted to all assay tubes. An aliquot of 50 *μ*l of mouse cytokines standard dilutions or serum to the tubes. The mixtures were incubated for 2 hr at room temperature, protected from light. Add 1 ml wash buffer to each assay tube and centrifuge at 200 *g* for 5 min. Carefully aspirate the supernatant from each assay tube and discard. Add 300 *μ*l of wash buffer to each assay tube to resuspend the bead pellet. Add 50 *μ*l of each of the following to the wells in the filter plate and cover the plate and shake it for 5 min at 1,100 rpm on a plate shaker. Add 120 *μ*l of wash buffer to each well to resuspend the beads and cover the plate and shake it for 2 min at 1,100 rpm before collecting samples. Analyze cytokine data using FCAP Array software.

### 2.8. Statistical Analysis

Employing SPSS v.20.0, data were analyzed by one-way analysis of variance (ANOVA) as well as Student's *t*-test. The least significant difference approach was employed for determining the variation across the groups, and the significance threshold was set at *P* < 0.05. The outcomes were shown as mean ± standard deviation.

## 3. Results

### 3.1. Screening and Validation of DMF as an Effective Hit for Inhibiting NO Production in RAW Cells

We screened a subset of the Johns Hopkins Drug Library in an LPS-stimulated NO production assay using RAW264.7 cells and DMF emerged as one of the most interesting hits. In response to LPS stimulation, DMF inhibited LPS-stimulated NO production in RAW264.7 cells in a dose-dependent manner (Figure 1(b)). Next, we determined the effect of DMF on the level of iNOS (inducible nitric oxide synthase), whose expression has been reported to be upregulated by LPS signaling. We observed that, similar to NO, LPS-induced iNOS expression is also inhibited by DMF in a dose-dependent manner, suggesting that the inhibition of NO by DMF is likely secondary to its inhibition of iNOS expression (Figure 1(c)).

### 3.2. DMF's Effects on the Production of Inflammatory Cytokines

**Figure 2:**
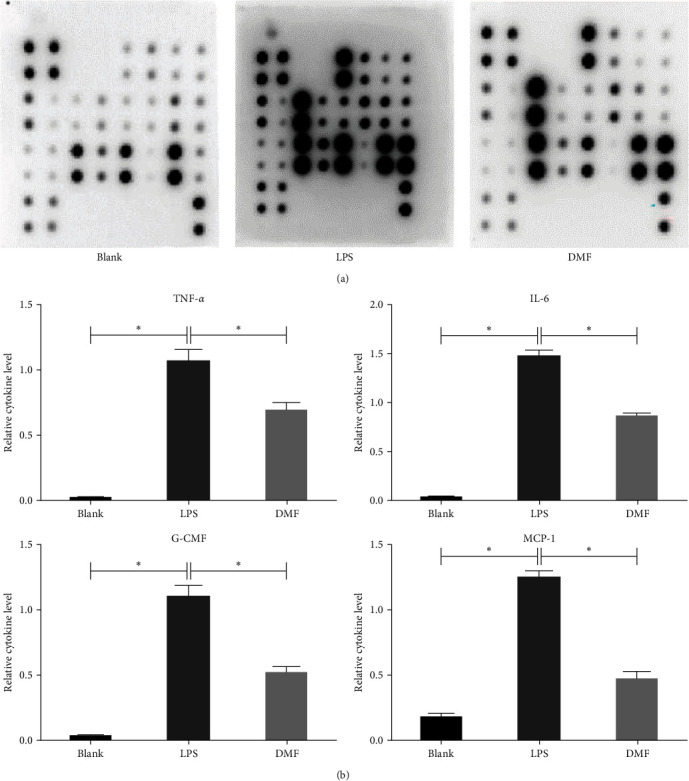
Effects of DMF on TNF-*α*, IL-6, G-CMF, and MCP-1 production by LPS-activated murine macrophages. RAW264.7 cells underwent incubation with DMF and subsequent treatment with 10 *μ*g/ml LPS. (a) Cytokine array panel was represented by the membrane. The production of several cytokines was shown by rounded black spots, and the gray value of each cytokine was determined. (b) The results indicated that, compared with the blank group, the expressions of TNF-a, IL-6, G-CMF, and MCP-1 were significantly increased after LPS stimulation. However, after DMF administration, the gray values of TNF-a, IL-6, G-CMF, and MCP-1 were decreased, respectively, compared with LPS control. The values are presented as the mean ± SD of three different samples. The student's *t*-test was employed for determining statistical significance.  ^*∗*^*P* < 0.05.

We determined the effect of DMF on the production of inflammatory cytokines in response to LSP treatment using a Mouse Cytokine Array Panel. As shown in Figure 2(a), after LPS stimulation of RAW264.7 cells, the expression levels of several cytokines increased among the 40 cytokines monitored. Upon DMF treatment, the protein levels of various cytokines were significantly decreased in comparison to the control. Among them, TNF-*α*, G-CMF, IL-6, and MCP-1 decreased appreciably compared with the LPS control group (Figure 2(b)).

### 3.3. DMF Protected against Mortality and Attenuated Excessive Inflammation in Septic Mice

**Figure 3:**
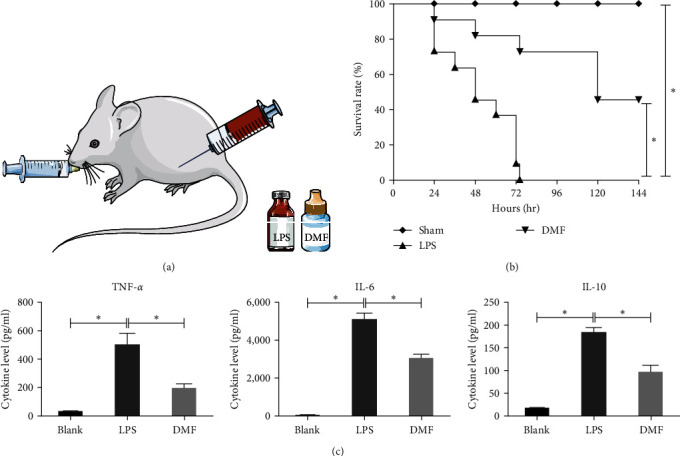
DMF protected against mortality and attenuated excessive inflammation in septic mice. C57BL/6 mice were administered DMF (30 mg/kg, gavage) or vehicle (saline) 30 min through injection before LPS injection (20 mg/kg, i.p.), survival was noted at various times, and the sera were collected. (a) The model diagram of the animal experiment is shown. (b) The log-rank test was employed to evaluate the statistical significance of survival rate. The mice had 100% mortality at 74 hr in the sepsis model. Treatment with DMF before the LPS challenge could increase the survival rate of mice by 50% compared with the sepsis model group. (c) Mouse cytokine array panel was to assess the generation of TNF-*α*, IL-6, and IL-10. The cytokines were inhibited after DMF administion in comparison with the LPS control group. The data are presented as the mean ± SD of three independent samples. The student's *t*-test determined the statistical significance.  ^*∗*^*P* < 0.05.

To assess the effect of DMF on sepsis in vivo, we developed a mouse model of severe sepsis. The mice had 100% mortality at 74 hr after LPS injection. Treatment with DMF prior to the LPS challenge increased the survival rate by 50% compared with the control group (Figure 3(b)). We also detected the levels of inflammatory factors in the serum of mice by using mouse cytokine array panels in each group at 6, 12, and 24 hr after the intervention. The expression levels of IL-6, TNF-*α*, and IL-10 were found to be substantially higher in septic mice as expected [16]. The highest concentration of TNF-*α* and IL-10 was observed at 6 hr post-LPS injection, while the highest concentration of IL-6 appeared at 12 hr. Importantly, treatment with DMF significantly inhibited the upregulation of IL-6, TNF-*α*, and IL-10 throughout the course of the experiments (Figure 3(c)).

### 3.4. DMF Attenuated Multiple Organ Injury in LPS-Induced Sepsis

**Figure 4:**
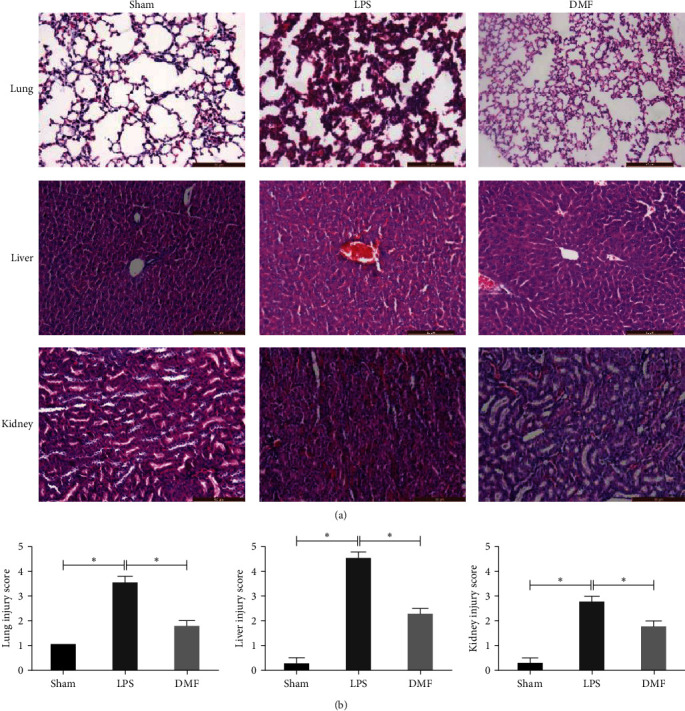
DMF attenuated multiple organ injury in LPS-induced sepsis mice. After sepsis mouse modeling and DMF treatment, different organ tissues were harvested 24 hr after LPS injection. (a) The outcomes demonstrated H&E staining of the lung, liver, and kidney tissue sections from the specified group. (b) The injury scores of lung, liver, and kidney were shown in the figure. The results show that DMF could significantly reduce the damage of lung, liver, and kidney tissue caused by sepsis, and the results are shown as the mean ± SEM for each. The figure represents three independent experiments.  ^*∗*^*P* < 0.05.

A hallmark of sepsis is multiple organ injury. We performed histopathological analyses of mice treated with DMF at 24 hr post-LPs injection. Tissue sections of the lung, kidney, and liver were stained with H&E. Upon LPS-induced sepsis, the lung, liver, and kidney all had significant injury that are manifested as inflammatory cell infiltration, hemorrhage, and cell death (Figure 4). The administration of DMF significantly alleviated the organ tissue damage of the lung, liver, and kidney, and the pathological scores of each organ were improved compared with those of the septic mice.

### 3.5. Effects of DMF on LPS-Induced Activation of the NF-*κ*B Signaling Pathway

**Figure 5:**
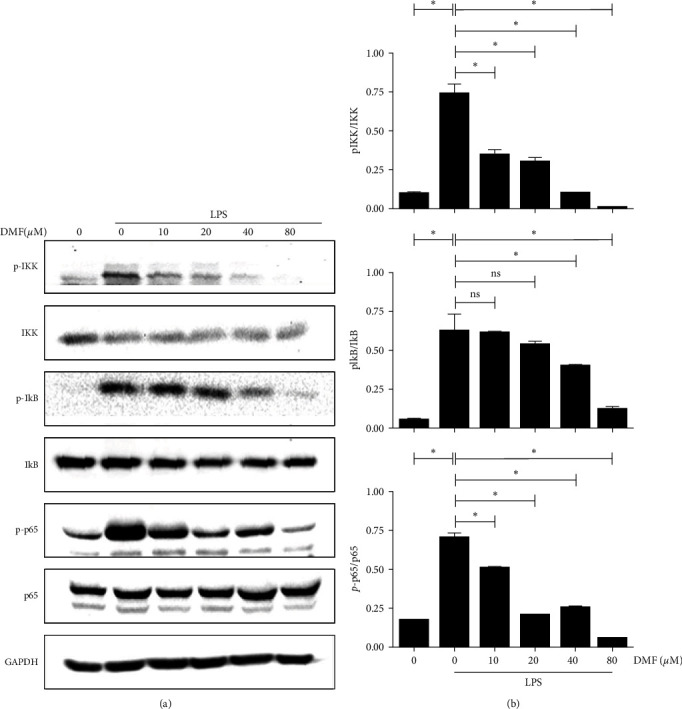
Effects of DMF on the LPS-induced NF-*κ*B signaling pathway. RAW264.7 cells were pretreated with varying concentrations of DMF before stimulation with LPS. (a) The cell signaling proteins, NF-*κ*B p65, I*κ*B, IKK, and their phosphorylated forms, were detected by western blot analysis with appropriate antibodies. (b) The protein levels of pIKK, IKK, pI*κ*B, I*κ*B, p- p65, and p65 were statistical analyzed by semi-quantitation method. On x-aixs, the first 0 corresponds to the blank group and the second 0 corresponds to the LPS stimulation group (without DMF). The values represent the mean ± SD of three separate experiments and Student's *t*-test was utilized for evaluating the differences in the mean values.  ^*∗*^Suggests significant difference between the indicated two groups (*P* < 0.05).

The transcriptional activation of iNOS expression by LPS requires the activation of NF-*κ*B among other transcription factors [16]. It has been reported that DMF is capable of inhibiting the phosphorylation as well as nuclear translocation of the p65 subunit of NF-*κ*B during dendritic cell maturation [20]. We thus determined the effects of DMF on LPS-stimulated NF-*κ*B activation. DMF inhibited the phosphorylation of p65 (Figure 5), similar to what was observed in dendritic cells. Aside from p65, however, we found that the phosphorylation of I*κ*B, as well as IKK in response to LPS treatment was also inhibited by DMF (Figure 5), suggesting that the site of action of DMF in LPS-stimulated RAW267.4 macrophage line lies upstream of p65. Together, these findings indicated that DMF's inhibitory effect on NO production as well as inflammatory cytokine production can be attributed to the suppression of NF-*κ*B activation by LPS through inhibition of a target that lies upstream of IKK.

## 4. Discussion

Sepsis is among the most severe complications and causes of mortality, leading to over 8 million deaths annually worldwide [13, 21]. Despite extensive clinical and basic research that has led to some progress in the management of several areas of sepsis, the therapeutic efficacy of existing drugs against sepsis remains limited. The current clinical treatment of sepsis is still mainly supportive treatment, which requires a lot of resources such as intensive care for patients, but can only provide limited efficacy.

Pathologically, sepsis leads to fatal organ dysfunction resulting from a dysregulated response to infection [13, 22]. Mechanistically, sepsis is typically initiated and driven by various pathogen-related molecular patterns (PAMPs), which are released by pathogenic microorganisms and recognized by the host's receptors, eventually leading to tissue and organ damage or failure, and deaths. In sepsis, bacterial-LPS endotoxin can be recognized by TLR whose activation induces macrophages to release large amounts of proinflammatory cytokines, causing cytokine storms [9, 23]. These proinflammatory factors can cause damage to the structure and activity of important organs such as the lung, liver, and kidney, culminating in multiple organ dysfunction syndrome (MODS) and eventual mortality. Hence, inhibiting the release of proinflammatory cytokines during sepsis is considered an important strategy for treating sepsis and improving survival.

NO is known as an important inflammatory mediator. Upregulation of NO by activated macrophages in response to certain stimuli has been implicated in inflammation, cytotoxicity, and immune disorders [24]. Therefore, inhibition of NO production can be an effective way to reduce inflammation. By screening a subset of the Johns Hopkins Drug Library for inhibitors of LPS-stimulated NO synthesis in RAW264.7 cells, we found that DMF to be a promising anti-inflammatory agent (Figure 1(a)). In the follow-up experiments, we found that DMF is capable of inhibiting LPS-stimulated NO synthesis in a dose-dependent manner (Figure 1(b)). Furthermore, we found that the inhibitory effect of DMF on NO synthesis was secondary to the reduction of iNOS expression (Figure 1(c)).

Macrophages are important participants and regulatory cells in sepsis-related inflammatory responses [12]. Macrophages can recognize LPS released by Gram-negative bacteria through TLRs, leading to excessive expression of inflammatory factors, including IL-1*β*, TNF-*α*, and IL-6, which are considered the leading cause of multiple organ dysfunction in the early stage of sepsis [25]. In the present work, we found that DMF is a novel inhibitor of LPS-induced NO synthesis. Importantly, we found that DMF also inhibited the LPS-induced cytokine production in LPS-stimulated RAW264.7 cells, including IL-6, G-CMF, TNF-*α*, and MCP-1 (Figure 2), suggesting that DMF may have the potential to treat various inflammation-related diseases including sepsis.

DMF not only inhibited LPS-induced NO and proinflammatory cytokine production in vitro, but also worked in vivo in a murine sepsis model. We found that DMF significantly reduced the mortality of LPS-induced sepsis mice. The tissue damage and inflammatory cell infiltration of vital organs such as lung, liver, and kidney were significantly reduced in DMF-treated septic mice, suggesting that DMF could alleviate the pathological damage of organs caused by LPS (Figure 4).

DMF has shown potent anti-inflammatory effects. It has been reported that DMF exerts potential anti-inflammatory and antioxidative effects through the nuclear factor erythroid 2-related factor 2 pathway as well as NF-*κ*B nuclear translocation [26–28]. NF-*κ*B is a major transcriptional factor in modulating the immune and inflammatory responses [29]. There are five NF-*κ*B members in mammalian cells, p50, p52, RelA (p65), c-Rel, and RelB, with the best characterized being the p50/p65 heterodimer [30]. In most cell types, NF-*κ*B complexes are present in an inactive state sequestered in the cytoplasm by its inhibitor I*κ*B [31]. Upon activation of TLRs by LPS, IKK undergoes phosphorylation, which in turn phosphorylates I*κ*B, leading to its ubiquitination and degradation by the proteasome, releasing the bound NF-*κ*B complex. Free NF-*κ*B subsequently translocates into the nucleus where it initiates transcriptional activation of its target genes. The primary mode of inhibition of NF-*κ*B by DMF has been reported to be caused by its inhibition of p65 phosphorylation in dendritic cells [20, 32, 33]. In contrast, we observed that DMF inhibited the phosphorylation of IKK in a dose-dependent manner, suggesting that the site of action of DMF in macrophages lies upstream of IKK. The precise molecular target remains to be identified.

Although our study demonstrates that DMF could inhibit NO and proinflammatory cytokine production in response to LPS and improve survival in LPS-induced septic mice, several limitations emerge from our study. Sepsis can cause complex pathological and physiological processes, of which LPS-induced endotoxemia is an important aspect. Due to the varied nature of sepsis, it is unlikely that LPS-induced endotoxemia model involved in our study could mimic all aspects of the clinical and biological complexity of the disease encountered in humans. Therefore, in the future, we can detect the role of DMF in other animal models that better reflect sepsis, in order to facilitate the future clinical application of this small molecule drug.

## 5. Conclusion

DMF has been used as a treatment for MS and other diseases for a long time. It has been used in the chronic settings in MS patients and is well-tolerated. The results presented in this study raise the exciting possibility that it may be effective in treating sepsis through its inhibition of the LPS-induced NF-*κ*B signaling pathway in macrophages.

## Data Availability

The data presented in this study are available upon reasonable request from the corresponding author.

## References

[1] Schett G., Neurath M. F. (2018). Resolution of chronic inflammatory disease: universal and tissue-specific concepts. *Nature Communications*.

[2] Serhan C. N., Savill J. (2005). Resolution of inflammation: the beginning programs the end. *Nature Immunology*.

[3] Iwasaki A., Medzhitov R. (2004). Toll-like receptor control of the adaptive immune responses. *Nature Immunology*.

[4] Lin C. K., Kazmierczak B. I. (2017). Inflammation: a double-edged sword in the response to *Pseudomonas aeruginosa* infection. *Journal of Innate Immunity*.

[5] Calvano S. E., Xiao W., Richards D. R. (2005). A network-based analysis of systemic inflammation in humans. *Nature*.

[6] Coussens L. M., Werb Z. (2002). Inflammation and cancer. *Nature*.

[7] Brasier A. R. (2018). Therapeutic targets for inflammation-mediated airway remodeling in chronic lung disease. *Expert Review of Respiratory Medicine*.

[8] Rhodes A., Evans L. E., Alhazzani W. (2017). Surviving sepsis campaign: international guidelines for management of sepsis and septic shock: 2016. *Critical Care Medicine*.

[9] Chousterman B. G., Swirski F. K., Weber G. F. (2017). Cytokine storm and sepsis disease pathogenesis. *Seminars in Immunopathology*.

[10] Tu Z., Zhong Y., Hu H. (2022). Design of therapeutic biomaterials to control inflammation. *Nature Reviews Materials*.

[11] van der Poll T., van de Veerdonk F. L., Scicluna B. P., Netea M. G. (2017). The immunopathology of sepsis and potential therapeutic targets. *Nature Reviews Immunology*.

[12] Galli G., Saleh M. (2021). Immunometabolism of macrophages in bacterial infections. *Frontiers in Cellular and Infection Microbiology*.

[13] Evans L., Rhodes A., Alhazzani W. (2021). Surviving sepsis campaign: international guidelines for management of sepsis and septic shock 2021. *Intensive Care Medicine*.

[14] Lu Y.-C., Yeh W.-C., Ohashi P. S. (2008). LPS/TLR4 signal transduction pathway. *Cytokine (Philadelphia, Pa.)*.

[15] Rosadini C. V., Kagan J. C. (2017). Early innate immune responses to bacterial LPS. *Current Opinion in Immunology*.

[16] Kim Y.-S., Ahn C.-B., Je J.-Y. (2016). Anti-inflammatory action of high molecular weight Mytilus edulis hydrolysates fraction in LPS-induced RAW264.7 macrophage via NF-*κ*B and MAPK pathways. *Food Chemistry*.

[17] Kornberg M. D., Bhargava P., Kim P. M. (2018). Dimethyl fumarate targets GAPDH and aerobic glycolysis to modulate immunity. *Science*.

[18] Linker R. A., Haghikia A. (2016). Dimethyl fumarate in multiple sclerosis: latest developments, evidence and place in therapy. *Therapeutic Advances in Chronic Disease*.

[19] Li H., Wang S., Zhan B. (2017). Therapeutic effect of *Schistosoma japonicum* cystatin on bacterial sepsis in mice. *Parasites & Vectors*.

[20] Peng H., Guerau-de-Arellano M., Mehta V. B. (2012). Dimethyl fumarate inhibits dendritic cell maturation via nuclear factor *κ*B (NF-*κ*B) and extracellular signal-regulated kinase 1 and 2 (ERK1/2) and mitogen stress-activated kinase 1 (MSK1) signaling. *Journal of Biological Chemistry*.

[21] Rhee C., Dantes R., Epstein L. (2017). Incidence and trends of sepsis in US hospitals using clinical vs claims data, 2009–2014. *Journal of the American Medical Association*.

[22] Hagel S., Bach F., Brenner T. (2022). Effect of therapeutic drug monitoring-based dose optimization of piperacillin/tazobactam on sepsis-related organ dysfunction in patients with sepsis: a randomized controlled trial. *Intensive Care Medicine*.

[23] Cavaillon J.-M. (2018). Exotoxins and endotoxins: inducers of inflammatory cytokines. *Toxicon*.

[24] Mo C., Wang L., Zhang J. (2014). The crosstalk between Nrf2 and AMPK signal pathways is important for the anti-inflammatory effect of berberine in LPS-stimulated macrophages and endotoxin-shocked mice. *Antioxidants & Redox Signaling*.

[25] Ge Y., Xu X., Liang Q., Xu Y., Huang M. (2019). *α*-Mangostin suppresses NLRP3 inflammasome activation via promoting autophagy in LPS-stimulated murine macrophages and protects against CLP-induced sepsis in mice. *Inflammation Research*.

[26] Scannevin R. H., Chollate S., Jung M.-Y. (2012). Fumarates promote cytoprotection of central nervous system cells against oxidative stress via the nuclear factor (erythroid-derived 2)-like 2 pathway. *Journal of Pharmacology and Experimental Therapeutics*.

[27] Ashrafian H., Czibik G., Bellahcene M. (2012). Fumarate is cardioprotective via activation of the Nrf2 antioxidant pathway. *Cell Metabolism*.

[28] Schulze-Topphoff U., Varrin-Doyer M., Pekarek K. (2016). Dimethyl fumarate treatment induces adaptive and innate immune modulation independent of Nrf2. *Proceedings of The National Academy of Sciences of the USA*.

[29] Choi M.-C., Jo J., Park J., Kang H. K., Park Y. (2019). NF-*κ*B signaling pathways in osteoarthritic cartilage destruction. *Cells*.

[30] Gilmore T. D. (2006). Introduction to NF-*κ*B: players, pathways, perspectives. *Oncogene*.

[31] Lai J.-L., Liu Y.-H., Liu C. (2017). Indirubin inhibits LPS-induced inflammation via TLR4 abrogation mediated by the NF-kB and MAPK signaling pathways. *Inflammation*.

[32] Loewe R., Holnthoner W., Groger M. (2002). Dimethylfumarate inhibits TNF-induced nuclear entry of NF-*κ*B/p65 in human endothelial cells. *Journal Of Immunology*.

[33] Haarmann A., Nehen M., Deiß A., Buttmann M. (2015). Fumaric acid esters do not reduce inflammatory NF-*κ*B/p65 nuclear translocation, ICAM-1 expression and T-Cell adhesiveness of human brain microvascular endothelial cells. *International Journal of Molecular Sciences*.

